# Clinical and Epidemiological Features of Hospitalized and Ambulatory Patients with Human Monkeypox Infection: A Retrospective Observational Study in Portugal

**DOI:** 10.3390/idr14060083

**Published:** 2022-10-27

**Authors:** João Caria, Raquel Pinto, Ema Leal, Vasco Almeida, Gonçalo Cristóvão, Ana Catarina Gonçalves, Margarida Torres, Maria Beatriz Santos, Hélder Pinheiro, Diana Póvoas, Diana Seixas, Sara Lino, Orlando Cardoso, Maria José Manata, Ana Virgolino, Fernando Maltez

**Affiliations:** 1 Departamento de Doenças Infeciosas, Hospital de Curry Cabral, Centro Hospitalar de Lisboa Central, 1069-166 Lisboa, Portugal; 2 Departamento de Medicina Interna, Hospital do Espírito Santo, Ilha Terceira, 9700-049 Angra do Heroísmo, Portugal; 3 Faculdade de Ciências Médicas, Universidade Nova de Lisboa, 1169-056 Lisboa, Portugal; 4 Instituto de Saúde Ambiental (ISAMB), Faculdade de Medicina da Universidade de Lisboa, 1649-028 Lisboa, Portugal; 5 Laboratório Associado TERRA, Faculdade de Medicina da Universidade de Lisboa, 1649-028 Lisboa, Portugal

**Keywords:** monkeypox, outbreak, person-to-person transmission

## Abstract

Monkeypox, a neglected and re-emergent zoonotic disease caused by monkeypox virus (MPXV) infection, has been endemic in Central and Western Africa for decades. More recently, an outbreak has spread to a global level, occurring in sites with no previous reported cases and being clustered among men who have sex with men, suggesting new modes of transmission. There is an urgent need for research for a better understanding of the genomic evolution and changing epidemiology of the Orthopoxvirus group. Our work aimed to characterize the clinical and epidemiological features of a cohort of patients with MPXV infection in a Portuguese hospital, admitted between 5 May and 26 July 2022. In this retrospective observational study, aggregate data of a case series on the presentation, clinical course, and outcomes of confirmed MPXV infections are reported. The study included 40 men and 1 woman, with a mean age of 37.2 years old; 92.7% identified as men who have sex with men, 90.2% had unprotected sex or sex with multiple or anonymous partners in the previous month, and 39.0% reported to have had sex with an MPXV-confirmed case; 59.5% had previously known human immunodeficiency virus (HIV) infection, all of whom were under antiretroviral therapy, and no patients had acquired immunodeficiency syndrome (AIDS) criteria. About a quarter of patients were observed only a week after symptom onset. All patients had skin or mucosal lesions and the anogenital region was the most frequent lesion site. There were no statistically significant clinical differences between HIV-positive and negative individuals. Four patients were admitted to the inpatient clinic, two of whom had proctitis with difficult-to-manage anal pain. There were no reported deaths. Our findings suggest the sexual route as a relevant mode of transmission of MPXV and confirm the mostly benign presentation of this disease.

## 1. Introduction

Human disease by monkeypox virus (MPXV) infection is a viral zoonosis with symptoms similar to, though less severe, than those observed in smallpox. After the suspension of widespread smallpox vaccination programs that led to the global eradication of the disease in 1980 [1], over 70% of the world’s population is no longer protected against smallpox nor too closely related to Orthopoxviruses due to waning cross-immunity [2,3].

The MPXV was first isolated in 1958 from macaque skin lesions [4]. However, the first human monkeypox (MPX) case was only described in 1970, in the Democratic Republic of Congo, in an infant with smallpox-like eruptions [5]. Moreover, the MPXV reservoir is still unknown, even though rodents and other small mammals are suspected to play an important role in the transmission in endemic sub-Saharan African countries [6,7]. The typical clinical syndrome is characterized by a febrile prodromal period of 1–4 days, associated with headache and fatigue, followed by a centrifugal development of a rash similar to smallpox, which evolves sequentially from macular-papular to vesicular, pustular (umbilicated lesions), and finally crusted scab lesions. Unlike smallpox, lymphadenopathy is more frequent in MPX and may develop before or concurrently with the rash [8]. The incubation period can vary between 5 and 21 days, with an average of 6 to 16 days, although there are reported cases with a maximum incubation period of 34 days [9].

The spectrum of the disease varies from mild to severe, with case fatality rates ranging from 1 to 10% [10]. Its severity is closely correlated to two distinct genetic clades of the MPXV: clade I (the former central African (Congo Basin) clade), more virulent, with greater mortality and more transmissible; and clade II (the former West African clade) [11,12,13]. Smallpox-unvaccinated people appear to be more susceptible and severe cases (including death) tend to occur more commonly among children, being related to the extent of virus exposure, patient health status, and nature of the complications [14]. Related immune deficiencies such as human immunodeficiency virus (HIV) infection may lead to worse outcomes [15].

For several years, MPXV transmission has been restricted to remote rural locations at a human-animal interface within humid tropical forests in central and West Africa, and only occasionally were travel-associated outbreaks reported in other continents [7,16]. One example was the 2003 outbreak in the USA caused by rodents imported from Ghana that infected co-housed prairie dogs [17] or the 2018 outbreak in the United Kingdom (UK), where human-to-human transmission outside African endemic countries was confirmed by a hospital hygiene officer probably after contact with soiled bedsheets [18]. Furthermore, some imported cases were reported between 2018 and 2021 in the UK, Israel, Singapore, and the United States of America (USA), and all were related to infected patients that travelled to these countries [16].

Against this background, the scientific community is raising questions about a putative change in the epidemiological pattern of outbreaks as well as in the biology of the virus [19]. While close physical contact is a well-known risk factor for transmission, other important aspects such as the clinical significance of long-term viremia and upper respiratory tract viral shedding, the importance of the sexual transmission route, the uncertainty regarding the risk of vertical transmission, or the prevalence of asymptomatic infection are still controversial. Thus, up-to-date research is needed to guide timely response in infection control and outbreak response strategies.

On 23 July 2022, the World Health Organization (WHO) declared the multi-country outbreak of MPX a Public Health Emergency of International Concern, highlighting that between 1 January and 2 July 2022, 25,022 cases were confirmed, including nine deaths, in 83 countries, mostly in Europe and the USA. Most cases currently occur among males who identify themselves as men who have sex with men (MSM), in urban areas and are clustered in social and sexual networks.

In Portugal, the first cases were presented in sexually transmitted diseases clinics in Lisbon on 3 May and the molecular confirmation (with MPXV nucleic acid amplification testing (NAAT)) of the first cases happened on 17 May [20]. Nowadays there is sustained human-to-human transmission of MPXV in Portugal, with more than 800 documented cases. Within this context, a better characterization of the disease is still missing to inform targeted public health measures that could prevent the dissemination of the MPXV, especially among the most vulnerable groups.

This study aimed to characterize the clinical and epidemiological features of a cohort of patients with monkeypox infection followed in a Portuguese Hospital.

## 2. Materials and Methods

The study followed a retrospective observational cross-sectional study with a purposive sample of patients admitted to the Infectious Disease Department of the Hospital of Curry Cabral in Lisbon, Portugal.

### 2.1. Setting and Participants

We analysed the medical records of all patients with a laboratory-confirmed positive result for monkeypox infection, admitted to the Infectious Disease Department of the Hospital of Curry Cabral in Lisbon, Portugal, either in the out or inpatient setting, between 5 May and 26 July 2022. Confirmed cases were defined by a positive MPXV NAAT from any anatomical site. We excluded all patients without laboratory confirmation of MPXV infection.

### 2.2. Variables

Demographic and epidemiological data including age, gender identity, sexual orientation, HIV infection, travelling history, probable transmission route, attendance of sex on-site venues, attendance of large events and chemsex use, and clinical data including presenting symptoms and signs, complications of the disease, treatments received, and laboratory results, were obtained as part of routine patient care and are presented here as aggregate data.

MPXV NAAT was performed in the Portuguese national reference laboratory, Instituto Nacional de Saúde Doutor Ricardo Jorge. Collected samples included urine, swabs of skin lesion fluid, and nasopharyngeal swabs, which were taken according to clinical symptoms by either a doctor or nurse. The samples were stored in the Clinical Pathology department of the Centro Hospitalar de Lisboa Central and sent to Instituto Nacional de Saúde Doutor Ricardo Jorge (Portugal) every weekday following the standard storing procedures.

### 2.3. Data Analysis

Statistical analysis was done with SPSS 27.0.1.0. For quantitative variables, we present mean, standard deviation, median, and interquartile range, and for categorical variables, we present absolute and relative frequencies. Hypothesis testing was performed using T-student and Chi-square tests (or Fisher exact test, when appropriate, for categorical variables), and statistical significance was considered for *p* < 0.05.

### 2.4. Ethical Issues

Patients included in this analysis signed written informed consent for the publication of anonymized clinical information and the publication of clinical images. Only relevant epidemiological and clinical data were collected, limiting the description of personal details. Data were de-identified by assigning a code to each subject and creating a separate identification database that was destroyed after data collection.

## 3. Results

We report the findings relative to a total of 41 patients with confirmed MPXV infection. Demographic, epidemiological, and clinical data of the patients with MPXV are summarized in Table 1, comparing HIV versus non-HIV-infected patients. HIV infection was known in 25 patients (59.5%). Age ranged from 22 to 58 years, with mean and median ages of 37.2 and 37.00 years old, respectively. The mean age was significantly higher among HIV patients (*p* = 0.013). Only one patient was a female, the second confirmed case of MPX in women in Portugal. There were no patients who self-identified as transgender or non-binary. Concerning the reported sexual orientation, apart from this woman who had sex with men (WSM) and two men who self-identified as men who had sex with men and women (MSMW), all other patients were male who self-identified as MSM. Most of the participants were of Brazilian (43.9%) or Portuguese (36.6%) origin. Recent international travel to countries in Europe (i.e., Germany, Spain, Belgium, and the UK), Asia (Israel), and the Americas (Dominican Republic) in the month preceding symptom onset was reported by 7 (17.1%) out of the 41 patients. HIV testing was performed in 81.3% of those with previously unknown or negative HIV status and all results were negative, while 59.5% had previously known HIV infection (data not displayed in the table). The most frequent concomitant sexually transmitted infection (STI) was gonorrhoea. Hepatitis C detectable viral load was present in three patients with known and untreated chronic infection by the hepatitis C virus. All but 4 patients reported sex with multiple and/or anonymous partners in the previous month and 16 of the subjects were sex contacts of MPX-confirmed cases. In the previous month, six individuals had attended sex parties or venues and eight reported having engaged in “chemsex” during that period. About a third was on HIV preexposure prophylaxis (PrEP). Only three patients had a history of smallpox vaccination.

Demographic and clinical data concerning HIV-infected patients are summarized in Table 2. The mean and median TCD4^+^ cell count of HIV-positive patients before monkeypox infection diagnosis were 776 and 702 cells/mm^3^, respectively, ranging from a minimum value of 244 to a maximum of 1728 cells/mm^3^. All 25 HIV-positive patients were on antiretroviral therapy (ART) and only 3 patients had a detectable viral load above 50 copies/mL. None of these three patients met the criteria for virological failure since they were all on ART for less than six months with a sustained decrease in viral load since the beginning of treatment. Most of the HIV-positive patients were on three-drug regimens, with the integrase inhibitor class as the most frequent third agent.

**Figure 1:**
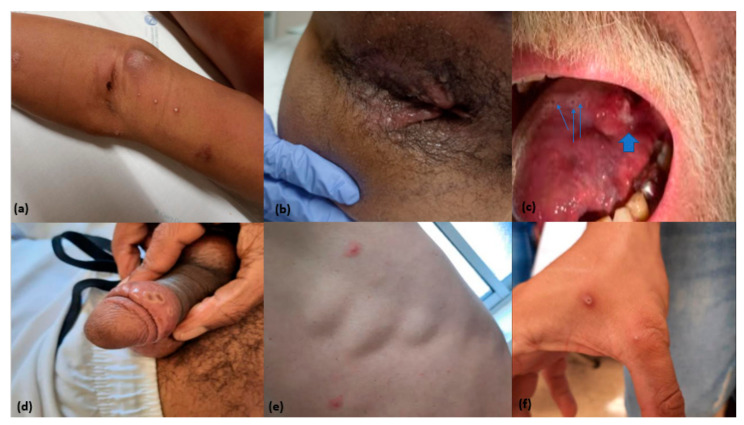
Skin and soft tissue manifestations of monkeypox in this case series. (**a**) Upper limb pustules and cicatrized lesion in venopunction site (needle sharing with confirmed case). Below the cicatrized puncture lesion there is an abscess with isolation of methicillin-resistant Staphylococcus aureus (patient B described in Table 4). (**b**) Anal umbilicated pustules and abundant exudation in a patient with proctitis (patient A described in Table 4). (**c**) Three umbilicated lesions (small arrows) and moderate size painful ulcer in left lateral tongue side—outpatient case. (**d**) Three umbilicated pustules in penis foreskin—outpatient case. (**e**) Two trunk lesions—in pustular and macular phases in a patient with diffuse trunk rash—outpatient case. (**f**) Two hand lesions—in pustular and umbilicated pustule phases in a patient with upper limb rash—outpatient case.

Aspects concerning the clinical presentation, diagnosis, and follow-up of MPX cases in this case series are summarized in Table 3. The Infectious Diseases Department where the research team of this study works was the first medical service that included patients resorted to in the context of MPX (78.0%). The average time between symptom onset and the first medical consult and testing was 6.6 days. There were cases of consult and testing on the same day of symptom onset and a maximum length of 28 days. In 9 out of 35 cases in which it was possible to determine the time interval between symptom onset and MPX screening, approximately a quarter (25.7%) were only tested one week after symptom onset. Regarding clinical features, all patients had skin lesions (some images of the skin or mucosal lesions of remarkable cases are displayed in Figure 1), while 78.0% of the patients presented a vesicular, macular, or pustular rash, 14.6% had a single ulcer, and 7.3% presented multiple ulcers. Although not statistically significant, there was a tendency for a less frequent presentation with vesicular, macular-papular, or pustular rash in the HIV subgroup versus the non-HIV subgroup of patients (32.0% vs. 6.2%; *p* = 0.052). The most common locations for the skin lesions were anogenital in the HIV subgroup of patients (64.0%), but the trunk and/or limb location was more frequent in seronegative patients (62.5%). Of note, almost one-third of the patients had face and/or mouth lesions (31.7%). Concerning the number of skin lesions shown, almost half of the patients (46.3%) had fewer than five skin and/or mucosal lesions, and only three had more than twenty lesions. The one patient that presented more MPX lesions had 61. Skin lesions were often in different evolution stages, with papules, centrally ulcerated umbilicated, or crusts simultaneously visible in the same patient. Apart from skin lesions, the other most common clinical features were fever (51.2%), adenopathies (46.3%), and odynophagia (26.8%), with no statistically significant differences between HIV and non-HIV patients. There were also no statistically significant differences regarding the average time from symptom onset to medical observation between the subgroup of patients with single ulcers versus the patients with other skin disease presentations (3.8 vs. 7.1 days). All patients had positive NAAT results for MPXV in lesion exudate; 34.1% had a positive result in oropharyngeal exudate, out of only a total of 30 patients tested for MPXV presence in the oropharynx, resulting in a positive test rate of 46.7%. Only one patient had detectable MPXV in the urine, of five tested patients.

All three smallpox-vaccinated patients were men. The information on vaccination was self-reported and none of these patients had documentation confirming the immunization date or regimen used. They were 44, 53, and 54 years old, respectively. Two of them were born in Brazil and the other in Cape Verde. All had a history of HIV infection and were on ART, two of them with long-time viral suppression while one with the last HIV-1 viral load of 66 copies/mL—this patient started ART less than six months before this determination. The last TCD4^+^ count of these patients was 345, 702, and 1728 cells/mm^3^. Clinically, one patient presented with a single inguinal adenopathy and less than ten lesions confined to the genital area with no further symptoms; another one with a single genital ulcer and with fever, inguinal adenopathy and pharyngitis; a third one presented with diffuse typical umbilicated lesions (>20) with the trunk, limb, and genital locations. Along with positive MPXV NAAT in skin/mucosal lesions, two of them had positive MPXV NAAT in oropharyngeal swabs. All these patients were managed in an outpatient setting, as no one developed complications.

All patients received supportive treatment, but only four of them required hospitalization. The provided medical care varied among the four patients: two with proctitis were admitted for pain management, due to multiple painful anal ulcers and anal bleeding; one patient required hospitalization due to concomitant hypoxic pneumonia without additional infectious agents being identified; and, finally, in one case, hospital admission was necessary for patient monitoring and isolation caused by acute mental impairment due to drug intoxication. The main case features of these inpatient cases are summarized in Table 4.

## 4. Discussion

Our findings are in accordance with the data in the literature describing that young MSM have been disproportionally affected by the ongoing MPXV outbreak [21,22]. The great majority of the patients reported having had sex either with anonymous or multiple partners or without condom use, during the known incubation period of the disease. Moreover, an important percentage of patients confirmed having had sexual partners in the month previous to the onset of the symptoms who, in the meantime, had been diagnosed with MPXV disease. Additionally, the fact that the anogenital area was the most common location site for skin lesions supports the sexual route as the major mode of transmission in this series. Sexual transmission, namely by direct contact of mucosal skin with infected genital fluids or lesion exudate, has already been previously suggested by studies that confirmed anogenital location to be frequent [21]. This fact, along with the previous documentation of MPXV in the semen of a high percentage of subjects in one study [23] and documented higher probability of anal lesions in individuals performing receptive anal sex [24] supports that this route of transmission may be the driving force in the current outbreak. These observations accord with the general understanding that very close contact, including sexual contact, has fueled the current outbreak [25,26].

The preferential anogenital location of the lesions is particularly interesting because it may suggest that direct contact of the lesion with the skin may be sufficient for transmission and anal sex may also facilitate this route. Additionally, the high number of patients with oral, lip, and face lesions equally suggests that the same mechanism through oral sex may occur. Although our findings suggest this mechanism to be plausible, it has not been described in previous monkeypox outbreaks. Furthermore, the patient admitted for anal pain management and bacterial superinfection, who, apart from sex, also reported needle sharing with a single anonymous sex partner, presented typical papules and pustules in venopunction sites. This fact is of utter importance because it once more suggests the development of local lesions by direct inoculation of a potentially infectious fluid. Nevertheless, in this particular case, there is no way to confirm whether the inoculated fluid was blood or lesion exudate contaminating the needle.

Still regarding epidemiological aspects, although most cases were of foreign origin, all of these patients were living in Portugal at the time of diagnosis and with active follow-ups in the outpatient unit where the patients were followed. International travel in the previous month was reported in 17.1% of the patients, none from known endemic areas. Some patients engaged in sex parties, similar to what was described in other case series [21,22]. Nevertheless, data indicate that more than an international phenomenon, transmission has probably occurred within the national region.

We hypothesized that presentation with single ulcers might delay patient awareness of the disease and medical attention seeking. Nevertheless, not only was the correlation not verified, but there was less delay in clinical observation in patients with such a presentation. It is possible that the fact that most of these patients had very painful single anal ulcers and potentially stigmatizing single facial lesions might have played a role in prompt seeking for medical observation.

The fact that in a quarter of patients it took more than a week since symptom onset for patients to seek medical services and therefore to receive personalized recommendations to avoid transmission raises concerns that conditions that allow rapid diagnosis, case isolation, and contact tracing are lacking.

The clinical features of our case series are similar to what has been described in other European countries [21,22]. All patients presented skin lesions, mostly in the anogenital region, trunk, and/or limbs. Half of the patients had less than five lesions and only three had more than twenty lesions (with one showing a maximum of sixty-one lesions). In a previously documented case series, from 2018 to 2021 [27], the number of lesions was higher (10–150 lesions), compared to what was observed in this study. This decrease in the number of lesions in the current 2022 outbreak seems to suggest that it can be associated with less symptomatic disease. It is important to mention that skin lesions were often non-synchronous, presenting at different stages in the same patient, which was also described in other countries [21,22]. Besides the skin lesions, fever and lymphadenopathy were present in half of the patients, as in other case series [21,22,27].

Clinical features in HIV-infected patients were similar to the non-HIV subgroup, as observed in other reports [21]. The typical presentation with vesicular, popular, or pustular rash was more frequent in the non-HIV subgroup. Although this was not a statistically significant result, at the moment when it is still unclear whether immunosuppression confers increased susceptibility to infection or more severe disease, the fact that our series has a higher proportion of HIV-coinfected patients is probably due to a selection bias—since these patients were already linked to care in our unit and naturally resorted to medical attention after noticing clinical manifestations—which does not probably reflect a real higher incidence of monkeypox in the HIV-positive population. Furthermore, there was no initial diagnosis of HIV infection in individuals with monkeypox, which does not favour the hypothesis of these patients have more severe disease.

All patients received a confirmatory diagnosis of monkeypox by a positive NAAT for MPXV in lesion exudate. Oropharyngeal swabs and urine samples were collected in 25 and 5 patients, respectively, with a lower positive test rate (46.7% and 20%, respectively) compared with skin or mucosal lesion exudate. These results are in line with what was reported in the largest case series [21] and suggest that the best sample for MPXV identification is lesion exudate.

In this study, there was no mortality, and the disease expression was mostly mild in the patients studied, similar to what has been described in other countries [19]. The need for hospitalization was low. In line with what was observed in two case series [19,20], the most common clinical reason for admission was the management of perianal or rectal pain. The four patients treated in our inpatient unit for rectal pain were considered to have the syndromic diagnosis of proctitis since they presented with symptoms that suggested inflammation of anorectal mucosa—namely anal pain, rectorragia, tenesmus, and anal exudate.

No serious complications were observed, although we highlight the case of patient C, who was admitted to our unit for lower-right lobe pneumonia, at the time of admission already in a late phase of monkeypox infection. Besides positive MPXV NAAT in oropharyngeal secretions, there was no evidence to link pulmonary involvement to monkeypox infection. This case might reflect a pulmonary bacterial superinfection as already described in the literature as a complication of monkeypox.

All patients received support treatment, and no antiviral therapy or post-exposure prophylaxis with vaccination was given to close contacts. As our patients were mostly young and the smallpox national vaccination program was suspended decades ago, there was a low likelihood of having an important percentage of vaccinated individuals. To our knowledge, only three patients had previously received smallpox vaccination. Statistical comparison with non-vaccinated patients was not possible due to the low number of vaccinated individuals, disallowing robust conclusions.

This study has some limitations that should be taken into consideration when interpreting the findings. First, it is an observational study with a small sample size, in which all participants were symptomatic, sought medical care, and had a positive MPXV NAAT test. Asymptomatic or paucisymptomatic patients could have been missed, which may lead to an overestimation of symptoms and disease severity. Second, a selection bias may also have hampered the results, as patients with HIV infection or using PrEP were already linked to care and more likely to seek medical care in our unit.

## 5. Conclusions

In conclusion, our study highlights the need to raise awareness about aspects of disease manifestations and transmission prevention, both in the general population and healthcare givers. In individuals with less symptomatic manifestations, symptoms may go unnoticed, contributing to further disease spreading. Our findings should also urge further investigation of transmission mechanisms since other studies have also been pointing out sexual transmission as an important driving force in this current outbreak. Additionally, the description of a patient known to have had contact with infected blood through needle sharing is according to previous concerns raised in the literature about parenteral transmission, especially in lab workers. Therefore, we believe that prevention recommendations for the population should include avoidance of endovenous drug paraphernalia sharing, as well as other items that may carry infected blood.

## Figures and Tables

**Table 1:** Demographic, epidemiological, and clinical data of the patients with MPXV.

	All Patients (N = 41)	HIV-Positive Patients (n = 25)	HIV-Negative Patients (n = 16)	*p*-Value ^a^
Age				
Mean (SD)—years	37.2 (8.3)	39.6 (8.1)	33.6 (7.6)	0.013
Median (IQR)—years	37.0 (12.0)	38.0 (12.0)	31.0 (8.0)	
Minimum–maximum	22–58	29–58	22–50	
Gender– No. (%)				
Male	40 (97.6)	25 (100.0)	15 (93.7)	
Female	1 (2.4)	0 (0.0)	1 (6.3)	
Patient self-reported sexual orientation—No. (%)				
MSM	38 (92.7)	25 (100.0)	13 (81.2)	
MSMW	2 (4.9)	0 (0.0)	2 (12.5)	
WSM	1 (2.4)	0 (0.0)	1 (6.3)	
Country of origin—No. (%)				
Brazil	18 (43.9)	13 (52.0)	5 (31.3)	
Portugal	15 (36.6)	7 (28.0)	8 (50.0)	
France	2 (4.9)	2 (8.0)	0 (0.0)	
Colombia	2 (4.9)	1 (4.0)	1 (6.3)	
Spain	1 (2.4)	0 (0.0)	1 (6.3)	
Peru	1 (2.4)	1 (4.0)	0 (0.0)	
Cape Verde	1 (2.4)	1 (4.0)	0 (0.0)	
Lebanon	1 (2.4)	0 (0.0)	1 (6.3)	
International travel in the preceding month (before symptom onset)—No. (%)	7 (17.1)	3 (12.0)	4 (25.0)	0.513
Country of travel—No. (%)				
Israel	2 (28.6)	0 (0.0)	2 (66.7)	
Germany	1 (14.3)	1 (33.3)	0 (0.0)	
Spain	1 (14.3)	0 (0.0)	1 (33.3)	
Belgium	1 (14.3)	1 (33.3)	0 (0.0)	
United Kingdom	1 (14.3)	0 (0.0)	1 (33.3)	
Dominican Republic	1 (14.3)	1 (33.3)	0 (0.0)	
STI screening—No. (%)	38 (92.7)	24 (96.0)	14 (87.5)	0.686
Concomitant STI diagnosis—No. (n/total screened = %)				
Gonorrhoea	5 (13.2)	4 (16.0)	1 (6.3)	
Chlamydia	2 (5.3)	0 (0.0)	2 (12.5)	
Syphilis	1 (2.6)	0 (0.0)	1 (6.3)	
Viral hepatitis infection—No. (%)				
Positive HCV RNA	3 (7.3)	1 (4.0)	2 (12.5)	
Positive HBV surface antigen	1 (2.4)	1 (4.0)	0 (0.0)	
PrEP use		^b^	12 (75.0)	
Sex with multiple and/or anonymous partners or unprotected sex in the previous month—No. (%)				
Yes	37 (90.2)	21 (84.0)	16 (100.0)	
No	4 (9.8)	4 (16.0)	0 (0.0)	
Sex with MPX-confirmed case	16 (39.0)	12 (48.0)	4 (25.0)	0.250
Sex party or venue attendance in the previous month—No. (%)	6 (14.6)	5 (20.0)	1 (6.3)	0.446
“Chemsex” in the previous month—No. (%)	8 (19.5)	4 (6.0)	4 (25.0)	0.760
Reported or registered history of smallpox vaccination—No. (%)	3 (7.3)	3 (12.0)	0 (0.0)	

^a^ Hypothesis testing (Chi-Square) was not used to compare variables in which there were registered frequencies of 0 or expected frequencies of less than 5. ^b^ HIV-infected patients are not eligible for PrEP. HBV—hepatitis B virus; HCV—hepatitis C virus; HIV—human immunodeficiency virus; IQR—interquartile range; MPX—monkeypox; MSM—men who have sex with men; MSMW—men who have sex with men and women; PrEP—preexposure prophylaxis; RNA—ribonucleic acid; SD—standard deviation; STI—sexual transmitted infection; WSM—women who have sex with men.

**Table 2:** Demographic and clinical of HIV-positive patients.

	HIV-Positive Patients (n = 25)
Last TCD4+ cell count before monkeypox diagnosis—cells/mm^3^	
Mean (SD)	776 (377.5)
Median (IQR)	702 (385.0)
Minimum–maximum	244–1728
Last HIV viral load before monkeypox diagnosis—copies/mL (%)	
<50	22 (88.0)
≥50	3 (12.0)
Reported adherence to ART—No. (%)	25 (100.0)
ART backbone—No. (%)	
ABC-based three-drug regimen	10 (40.0)
TDF-based three-drug regimen	6 (24.0)
TAF-based three-drug regimen	6 (24.0)
Two-drug regimen	2 (8.0)
Other ^a^	1 (4.0)
ART third agent—No. (%)	
INSTI	17 (68.0)
NNRTI	6 (24.0)
Boosted PI + INSTI	1 (4.0)
Boosted PI	1 (4.0)

^a^ Other category includes a single patient in a regimen of DTG+DRV/c. ABCs—abacavir; ART—antiretroviral therapy; HIV—human immunodeficiency virus; INSTI—integrase inhibitor; IQR—interquartile range; NNRTI—non-nucleoside reverse transcriptase inhibitor; PI—protease inhibitor; SD—standard deviation; TAF—tenofovir alafenamide; TDF—tenofovir disoproxil fumarate.

**Table 3:** Clinical features at first presentation, diagnosis, and follow-up of patients in this case series.

	All Patients (N = 41)	HIV-Positive Patients (n = 25)	HIV-Negative Patients (n = 16)	*p*-Value ^a^
Time between symptom onset and medical observation—days				
Mean (SD)	6.6 (6.4)	3.8 (5.1)	7.1 (7.8)	0.131
Median (IQR)	4.0 (4.0)	3.0 (4.0)	6.0 (9.0)	
Minimum–maximum	0–28			
Clinical features—No. (%)				
Skin lesions	41 (100.0)	25 (100.0)	16 (100.0)	
Fever	21 (51.2)	13 (52.0)	8 (50.0)	0.901
Adenopathy	19 (46.3)	13 (52.0)	6 (37.5)	0.364
Odynophagia	11 (26.8)	8 (32.0)	3 (18.8)	0.350
Asthenia	9 (22.0)	7 (28.0)	2 (12.5)	0.242
Myalgia	9 (22.0)	7 (28.0)	2 (12.5)	0.242
Headache	6 (14.6)	5 (20.0)	1 (6.3)	0.224
Proctitis	2 (4.9)	1 (4.0)	1 (6.3)	0.744
Type of skin—No. (%)				
Vesicular, macular, or pustular rash	32 (78.0)	17 (68.0)	15 (93.8)	
Single ulcer	6 (14.6)	5 (20.0)	1 (6.3)	0.052
Multiple ulcers	3 (7.3)	3 (12.0)	0 (0.0)	
Location of skin lesions—No. (%)				
Anogenital	25 (61.0)	16 (64.0)	9 (56.3)	0.620
Trunk and/or limbs	23 (56.1)	13 (53.0)	10 (62.5)	0.510
Face and/or mouth	13 (31.7)	7 (28.0)	6 (37.5)	0.524
Number of skin lesions—No. (%)				
<5	19 (46.3)	13 (53.0)	6 (37.5)	0.364
5–10	10 (24.4)	7 (28.0)	3 (18.8)	0.501
11–20	9 (22.0)	3 (12.0)	6 (37.5)	0.054
>20	3 (7.3)	2 (8.0)	1 (6.3)	0.834
MPXV NAAT performed by anatomic region—n (%)				
Skin or mucosal lesions exudate	41 (100.0)	25 (100.0)	16 (100.0)	
Oropharyngeal exudate	30 (73.2)	19 (76.0)	11 (68.8)	0.456
Urine	5 (12.2)	1 (4.0)	4 (25.0)	
Positive MPXV NAAT—No. (%; relative to total tests performed)				
Skin or mucosal lesions exudate	41 (100.0)	25 (100.0)	16 (100.0)	
Oropharyngeal exudate	14 (46.7)	8 (42.1)	6 (54.5)	0.087
Urine	1 (20)	1 (100.0)	0 (0.0)	
Medical care follow-up—No. (%)				
Outpatient	37 (90.2)	23 (92.0)	14 (87.5)	0.877
Inpatient	4 (9.8)	2 (8.0)	2 (12.5)	

^a^ Hypothesis testing (Chi-square) was not used to compare variables in these rows since there were registered frequencies of 0 or expected frequencies of less than 5. HIV—human immunodeficiency virus; IQR—interquartile range; MPXV—monkeypox virus; NAAT—nucleic acid amplification test; SD—standard deviation.

**Table 4:** Case features of the four inpatient cases.

Patient	Demographic Data	Epidemiology	Clinical Features	Diagnosis	Treatment and Outcome
A	42-years-old, Colombian man	MSM; sex contact with confirmed case six days before symptom onset; HIV-negative; unvaccinated for smallpox	Onset of face rash six days after sexual contact. Clinical evolution with the onset of proctitis (proctalgia, anal ulcers, rectorragia, and anal purulent exudate) and generalized macular-vesicular rash. Admitted in the inpatient setting 10 days after symptom onset for pain management.	Positive NAAT result in lesion fluid (no more samples collected)	Support treatment; discharged after eight days, recovered from pain with no complications
B	37-years-old, Brazilian man	MSM; sex contact and needle sharing for drug injection with confirmed case four days before symptom onset; HIV-positive under ART with preserved immune status	Started with macular-vesicular lesions nearby the venopuncture sites and proctitis (anal pain, tenesmus, and exudation) four days after contact with a positive case. Admitted to an inpatient setting for anal pain management. After the first week of inpatient, while having multiple body and anal lesions in the cicatrization phase, develops new trunk and limb macular-vesicular lesions.	Positive NAAT results in lesion fluid in the admission moment and later in new lesion testing	Antibiotic treatment for bacterial superinfection (isolation of MRSA) of arm venopunction lesion sites; discharged with anal pain control
C	41-years-old, Brazilian man	MSM; sex worker; no known contact with MPX case; HIV-negative; PrEP use	Started with macular-vesicular trunk rash two weeks before admission to the inpatient unit. In the second week of disease, developed multiple perianal lesions, with a crusted appearance. On admission day starts fever and pain in the lower-right region of the thorax. In the emergency department, it is documented fever, hypoxemia, and thorax radiography with lower-right lobar condensation infiltrate suggesting bacterial pneumonia. Lab work reveals leucocytosis with neutrophilia and C-reactive protein of 270 mg/L. Admitted as an inpatient for pneumonia antibiotic treatment.	Positive NAAT results both in lesion fluid and oropharyngeal secretions	Antibiotic treatment for presumptive bacterial pneumonia with no agent identification (haemocultures, viral multiplex and S. pneumonia, and Legionella spp. urinary antigen tests were negative); discharged after four days of inpatient with good clinical and radiological evolution. Monkeypox lesions were all crusted
D	44-years-old, Brazilian man	MSM; no known contact with MPX case; HIV-positive under ART with preserved immune status	Presented to the emergency department due to psychiatric symptoms related to acute methamphetamine consumption. Presence of three suggestive vesicular lesions confined to the right arm with no further MPX symptoms. Admitted as an inpatient for monitoring of mental clinical evolution and contact avoidance.	Positive NAAT results both in lesion fluid and oropharyngeal secretions	Antipsychotic treatment for psychiatric symptoms with good clinical evolution. Discharged after four days as an inpatient with all three monkeypox lesions crusted

ART—antiretroviral therapy, HIV—human immunodeficiency virus; MRSA—methicillin-resistant Staphylococcus aureus; MSM—sex who have sex with men; NAAT—nucleic acid amplification test.

## Data Availability

Data may be sorted by contact with corresponding authors.
